# VCT clinic HIV burden and its link with HIV care clinic at the University of Gondar hospital

**DOI:** 10.1186/1471-2458-12-1010

**Published:** 2012-11-21

**Authors:** Getahun Asres Alemie, Shitaye Alemu Balcha

**Affiliations:** 1 Department of Epidemiology and Biostatistics, CMHS, University of Gondar, Gondar, Ethiopia; 2 Department of Internal Medicine, CMHS, University of Gondar, Gondar, Ethiopia

**Keywords:** Voluntary counselling and testing, HIV care, VCT/HIV care link

## Abstract

**Background:**

Voluntary Counselling and Testing (VCT) is an important component of any HIV/AIDS control and prevention activities. VCT makes people aware of their HIV serostatus and enables early identification of those who need care. It is an important link to HIV care and support. The main aim of this study is to describe the HIV burden at VCT and define the relationship between the VCT Center and the HIV Chronic Care Clinic of the University of Gondar (UoG) Hospital.

**Methods:**

It is a record based descriptive study undertaken by using data collected by health professionals at the VCT center and the HIV chronic care clinic of the UoG Hospital. Patient data collected from 2005/06 to 2008/09 was investigated. Analysis was carried out using the SPSS version 16.0.

**Results:**

A total of 19,168 people were tested for HIV and a prevalence of 25.4% was obtained. 4298 HIV positive people were referred to the HIV chronic care clinic but only 27% actually registered at the clinic. Chi-square analyses showed residence, age and time of VCT visit showed significant relations with hospital care attendance.

**Conclusion:**

The overall HIV prevalence is high. The data obtained at the HIV care clinic regarding patients’ clinical conditions at acceptance were incomplete. Improvements are required on the link between VCT and HIV care and documentation of data.

## Background

Decades have passed since HIV/AIDS started to be considered as a major threat in terms of its personal, family and socio-economic consequences. As a result HIV/AIDS has been investigated in many ways ranging from clinical to the public health aspects. Prevention, screening and counselling, stigma and discrimination, care to HIV patients and effectiveness and the complications of therapy are the focus areas. HIV counselling and testing is a major concern and the relationship between counseling and testing and HIV care is an important link to notice [1].

HIV voluntary counselling and testing (VCT) refers to the process of giving people professional counselling before and after the HIV test. VCT provides people with an opportunity to learn and accept their HIV serostatus in a confidential environment with counselling and referral for ongoing emotional support and medical care [2].

VCT is an entry point for HIV related programs. Knowledge of the quality of services and motivation for undergoing testing by individuals is important for effective understanding of the testing environment [3,4]. It is hence a valuable component of comprehensive HIV/AIDS programming. It is specifically important for individuals and couples to learn about their HIV status and make informed decisions about their future [4,5]. Additionally, VCT centers can be used for the estimation of the prevalence of HIV infection [6].

Provision of VCT centers, where people can seek out an HIV test, is helpful for those who are self motivated to be tested for HIV infection. Moreover, it is equally important to coordinate this center with other HIV related services. Early diagnosis of infected individuals and linkage to care are critical to improving an individual’s health and to secondary prevention efforts aimed at slowing the HIV epidemic [7].

A strong link between VCT and the HIV care clinic is vital in averting or decreasing untimely mortalities associated with HIV/AIDS showing the link is a critical structure for success in the treatment of people with HIV/AIDS. Moreover, VCT helps decrease late presentations and also assists PMTCT in the early diagnosis of HIV.

Amhara, Oromia, Addis Ababa and SNNPR (Southern Nations, Nationalities and People’s Region) account for 86.6% of the people living with HIV (PLWH) in Ethiopia. The overall prevalence of HIV in the Amhara region is 2.9%. This region is home for 31% of the PLWH in the country. According to the federal Ministry of Health, the region’s rural and urban prevalence rates are 1.5% and 9.9%, respectively. Gondar is among the biggest cities in the Amhara region. Antenatal care based assessments showed an HIV prevalence of 10.3%. Studies concerning HIV/AIDS will be of great benefit in considering the urgency of data needed for decision making [8-11].

Two things are of paramount importance when seeing the role of VCT in the prevention and control of HIV. The first is the overall prevalence of HIV positive patients at VCT. The second is how effectively the HIV positive patients are linked to the place where care is rendered. Therefore, the main aim of this study is to quantify the burden of HIV at VCT and see the link between the VCT center and the HIV care clinic of the UoG Hospital.

## Methods

This is a descriptive study conducted by reviewing the registration and charts available at the VCT center and HIV care clinic. The study looked at records of people who visited the VCT and HIV care clinics of the University of Gondar Hospital from 2005/06 to 2008/09 (An Ethiopian year spans from September to August; so for instance, 2005/06 covers September 2005 to August 2006).

The registration book at the VCT center was used to recruit all people who came to the center for an HIV test from 2005/06 to 2008/09. Data on some variables (client code, age, sex, address, religion, marital status, educational status, history of sexually transmitted diseases (STI) other than HIV/AIDS itself, HIV test result, etc.) was taken from the VCT registration book. The VCT registration book does not include second time HIV positive test results by the same persons. Individuals who came to the center many times and tested positive had their result registered only once. Using the client codes, HIV positive patients were traced in the HIV care clinic. Charts of the traced patients were used for data collation on the other variables (Past opportunistic infections, previous TB, functional status, WHO stage, need for evaluation, patient whereabouts, etc.). Data was taken from the registration books and charts from the period June to August 2010. Patients were checked at the HIV care clinic to see if they showed up there at any time after the HIV test at the VCT center until the time their charts were seen at the HIV care clinic.

The UoG Hospital is the biggest and the oldest referral hospital in the Amhara National Regional State of Ethiopia. This hospital, located in the heart of the historical city of Gondar, currently offers a variety of HIV related services to adults, pregnant women, children, etc. These services range from simple VCT to advanced laboratory supported care to patients with HIV/AIDS.

All visitors coming to and being tested at the VCT center of the hospital were the target population for the study. Records of all VCT visitors in the specified period of the study were retrieved from the VCT center and related records (if any) were also accessed from the HIV care clinic of the hospital by using patient names and VCT codes. VCT visitors whose HIV status was not stated in the registration book were automatically excluded from the study. Data registration forms (prepared by including the variables documented in the registration books and patient charts/ intake forms at the VCT center and HIV care clinic respectively) were used for collection of data. The data collection was undertaken in collaboration with health professionals working at the two centers (VCT center and HIV care clinic). Data collectors were oriented on how to access and retrieve information from the records. This data was then entered into a computer for analysis.

HIV serostatus, HIV care status (joined care or not), WHO clinical staging, socio-demographic variables (age, sex, marital status, educational status and religion) were used as the main variables in this study. The collected data was managed using SPSS version 16.0.

The study was conducted after ethical approval was obtained from the Institutional Review Board of the UoG and with the prior permission from the medical director’s office of the Hospital. The VCT center and HIV chronic care clinic were accessed with written permission from the medical director’s office. All record reviews were kept confidential.

## Results

A total of 19,168 adults visited and were tested for HIV at the VCT center of the UoG Hospital from 2005/06 to 2008/09. Four thousand eight hundred and sixty four individuals (25.4%) were found to be HIV positive. Out of these, 1359 (27.9% of the total positives), 1498 (30.8%), 1251 (25.7%) and 756 (15.5%) HIV positive patients were seen in the years 2005/06, 2006/07, 2007/08 and 2008/09, respectively. The year specific proportions of HIV positives were found to be 31.0%, 25.7%, 35.6% and 13.9% in the years 2005/06, 2006/07, 2007/08 and 2008/09, respectively.

Only the positive patients were studied to determine the link between the VCT center and the HIV chronic care. Tables 1 summarizes their socio-demographic characteristics.

**Table 1:** Sopcio-demographic characteristics of HIV positive patients seen at the VCT center of the UoG Hospital, 1998-2001 E.C.

**Socio-demographic variables**	**Frequency**	**Percentage**
Sex		
Male	2176	44.7
Female	2688	55.3
Residence		
Gondar	2529	52.0
Out of Gondar	2335	48.0
Religion		
Ethiopian Orthodox	4465	91.8
Islam	350	7.2
Protestant	27	0.6
Catholic	3	0.1
Others	19	0.4
Marital Status		
Single	1067	22.0
Married	1880	38.8
Divorced	1347	27.8
Widowed	549	11.3

The mean age of HIV infected patients was 32.1 years with a standard deviation of 8.9 years. The oldest age identified was 79 years. Most of the patients were found in their third or fourth decades. Out of all the HIV positive patients, 2688 (55.3%) were female patients. They were found to be a little younger than the male patients with Student’s t-test showing a statistically significant age difference (a mean difference of 3.8 years with 95% C.I of 3.2 to 4.4).

At the time of screening for HIV, the patients were asked if they had any history of sexually transmitted infections other than HIV in the past and 22% had some form of sexually transmitted disease. Close to half of the HIV positive patients (45.9%) felt sick at the time of screening. However, when asked about why they came for voluntary counselling and testing, 4224 (86.8%) did so because they wanted to know their HIV status. The availability and need for ART, marriage and reunion were the reasons for 583 (12.0%), 34 (0.7%) and 5 (0.1%) of the cases respectively.

Out of the total 4864 people who tested positive, 4298 (88.4%) were referred to the HIV chronic care clinic of the UoG Hospital while the rest were referred to other places based on their preferences. However, among patients referred to the chronic care clinic, only 1158 (26.9%) showed up and were documented at the clinic. Chi-square analyses were undertaken to obtain insight in the determinants for attending hospital care. The results of the analyses are shown in Table 2.

**Table 2:** Chi-square analysis showing the crude odds ratios and p-values for determinants of hospital care for HIV positive patients at the UoG Hospital, 1998 – 2001 E.C.

**Determinants**	**Hospital care**		**Crude odds ratio (95% C.I.)**	**Chi-square value (P-value)**
	**Yes**	**No**		
Sex				
Male	461	1451	R*	
Female	633	1689	1.14 (0.99 – 1.31)	P = 0.070
Residence				
Gondar	655	1752	R*	P = 0.003
Out of Gondar	439	1452	0.81 (0.70 – 0.93)	
Age				
15 – 24	147	623	R*	P < 0.001
25 – 44	828	2256	1.56 (1.28 – 1.89)	
45 – 64	113	315	1.52 (1.15 – 2.01)	
65 and above	6	10	2.54 (0.91 – 7.11)	
Time of VCT Visit**				
1998	276	976	R*	P < 0.001
1999	249	1216	0.72 (0.60 – 0.88)	
2000	347	713	1.72 (1.43 – 2.07)	
2001	222	299	2.63 (2.11 – 3.27)	

* R shows the reference category for comparison.

** Time of VCT Visit is according to the Ethiopian Calendar.

A number of assessments are routinely undertaken for new patients at the HIV chronic care clinic. In this study some were assessed using patient records showing presence of past opportunistic diseases, previous tuberculosis, functional status and WHO disease stage. In addition, the need for an evaluation of coughs/TB, diarrhoea, fever and prophylactic medications was assessed.

Out of the 1158 HIV positive patients who showed up at the HIV chronic care clinic, 824 (71.2%) had some form of opportunistic infection and 199 (17.2%) had TB (all had been treated for it) in the past. Out of those whose functional status at presentation was documented in their files (458 patients), 296 (64.6%), 151 (33.0%) and 11 (2.4%) were found to be working, ambulatory and bed ridden respectively. WHO staging was documented for 893 patients only and the majority (63.6%) had stage III HIV disease, followed by stage II (15.9%), stage IV (13.1%) and stage I (7.4%).

The need for evaluation of coughs/TB, diarrhoea and fever was documented for only 824, 816 and 860 patients respectively. Out of 824 patients with complete data, 194 (23.5%) needed evaluation for coughs/TB. Similarly, 25.6% and 40% of the patients with complete data needed evaluation for diarrhoea and fever respectively.

The need for prophylactic medications and anti-retroviral therapy (ART) eligibility was also assessed. The former was documented for only 705 patients (out of 1158 patients linked to the HIV chronic care clinic) who showed up at the clinic, while the later was done for only 762 patients. Six hundred fifty one patients (92.3%) needed prophylactic medication and 758 patients (99.5%) were found eligible for ART upon arrival at the HIV chronic care clinic. Regardless of poor documentation on eligibility, a total of 941 patients (81.3% of those who were linked to the chronic care clinic) were found to be on ART while the rest were being given pre-ART care at the time of the data collection. The percentages of those who were on ART were found to be 83.4% and 79.7% of male and female patients respectively.

## Discussion

In a setup where there is a high prevalence of HIV a VCT campaign is a very vital link to bring HIV infected patients to the healthcare services where they can get care and support. An HIV prevalence of 25.4% in this study is very high but comparable to some studies undertaken in urban Ethiopia [12,13]. With reference to the year specific figures, the first three years saw extremely high prevalence. The fourth year prevalence is comparable to the results of other VCT based studies in the country though higher than the national estimate [9,14]. The overall high prevalence we saw in this study may not be so surprising as we usually expect a relatively higher HIV prevalence in hospital based VCTs. The aftermath of the Ethiopian new millennium celebration that saw huge HIV testing campaigns throughout the country could be the reason for a reduced number of VCT in the year 2008/09 (Ethiopian year 2001). The same year was associated with intensified provider initiated HIV testing and counselling services in Gondar that might have led to the diagnosis HIV infected people at the care setting. This in turn would result in a reduced flow of HIV infected people to the hospital VCT center making that year’s HIV rate appear much lower than the other years.

The sex and age composition of HIV infected patients was assessed and women accounted for slightly more than half of all the cases. There was also a significant age difference of about 4 years between male and female cases. Women were found to be younger than men. This is in agreement with many studies which suggest the HIV epidemic is becoming more of a problem in women. The rate at which women are acquiring the disease is clearly higher than that of men [15-17].

An HIV diagnosis is not sufficient to ensure infected persons are able to access HIV treatment. Delay in showing up at HIV care clinics once diagnosed at the VCT centers leads to delayed initiation of antiretroviral therapy and prophylactic treatment potentially resulting in poorer prognosis and burdens to overstretched health care settings. So facilitating the link between HIV screening centers and care settings is a vital thing [18,19]. In this study, out of 4298 HIV infected patients referred to the HIV care clinic, only about 27% registered at the clinic. This is a very low number and the issue is a big public health concern in terms of the prevention and control of the HIV epidemic. A number of explanations can be given. Discussion between patients and health professionals working at the HIV care clinic might have led to the referral of patients to their nearest clinics thereby resulting in low registration of patients at the UoG Hospital HIV care clinic. On the other hand, patients might have escaped from the link on their own suggesting the need for more extensive work on the link between the VCT center and the HIV clinic.

Chi-square analysis showed that there was a statistically significant association between residence (p = 0.003), age (p < 0.001) and time of VCT visit (p < 0.001) and hospital care attendance. Patients from Gondar were 1.23 times more likely to attend hospital HIV care compared to those from outside Gondar. Living in Gondar seems to be a determining factor for attending care at the UoG Hospital. On the other hand, patients aged 25 and above were more likely to attend hospital HIV care than younger patients.

To view the overall picture more clearly, it is also wise to consider how the care referral system at UoG hospital operates in addition to the availability and the timing of decisions to start ART. UoG hospital has a very good clinic that provides follow up and standard treatment for people eligible for ART. However, there seems to be a problem in getting the patients to the care area. There is scarcity of counsellors and social workers who can physically take HIV positive people from the VCT clinic to the care clinic. The experience and effectiveness of health professionals at the VCT clinic might also be a concern for the poor link.

While the World Health Organization (WHO) has recommendations on the importance of specifically dedicated interventions to link up HIV tested individuals with care settings, the reality is that some settings are still having problems [20,21]. Studies have shown that there are places that are facing problems in linking HIV tested patients with HIV care settings. Uptake rates as low as 16% were seen in Tanzania [20].

Generally speaking the results of this study suggest that the burden of HIV positivity is high and most of the HIV infected patients who showed up at the HIV care clinic needed treatment and close follow up as their disease is advanced. If analysed together with the low number of registrations (27% for the overall and 25% for Gondar residents), one may conclude that the VCT center is behind in its role of linking patients to the HIV care clinic.

The absence of follow up for the HIV positive patients and their appearance at the HIV care clinic in the future and the fact that we have not used any advanced statistical analyses like Kaplan Meier to see the timing from testing to care entry can be considered a limitation of the study. Another limitation is that we did not see the delay time between VCT testing and entering into hospital care.

## Conclusions and recommendations

The study found a high prevalence of HIV especially in the first three years. The link between the VCT center and the HIV care clinic was found to be poor. The poor link suggests failure of VCT in acting as an entry point for further HIV care and treatment. Counselling sessions should be improved in order to ensure more patients are linked to the HIV care clinic. In addition, documentation at the care clinic was found to be poor and hence must be improved.

## Competing interests

The authors declare that they have no competing interests.

## Authors’ contributions

Dr. Getahun Asres Alemie and Dr. Shitaye Alemu Balcha have been actively involved in the data collection, data management, analysis and manuscript writing. Both authors read and approved the final manuscript.

## Pre-publication history

The pre-publication history for this paper can be accessed here:

http://www.biomedcentral.com/1471-2458/12/1010/prepub
